# Factors facilitating the use of contraceptive methods among urban adolescents and youth in Guinea: a qualitative study

**DOI:** 10.1186/s12978-023-01621-z

**Published:** 2023-06-13

**Authors:** Hawa Manet, Marie-Hélène Doucet, Charlotte Bangoura, Nafissatou Dioubaté, Alison M. El Ayadi, Sidikiba Sidibé, Tamba Mina Millimouno, Alexandre Delamou

**Affiliations:** 1 Maferinyah National Training and Research Center in Rural Health (CNFRSR), Forécariah, Guinea; 2 National Institute for Health and Medical Research (INSERM) UMR 1219, Research Institute for Sustainable Development (IRD) EMR 271, Bordeaux Population Health Centre, University of Bordeaux, Bordeaux, France; 3 Department of Obstetrics Gynecology and Reproductive Sciences, University of California San Francisco, San Francisco, USA; 4 African Centre of Excellence for the Prevention and Control of Communicable Diseases, University of Conakry, Conakry, Guinea

**Keywords:** Family planning, Adolescents and youth, Promoting factors, Urban, Guinea

## Abstract

**Background:**

The use of modern contraceptive methods among adolescents and youth is a public health priority to prevent unintended pregnancies. To our knowledge, no study has ever explored and documented factors promoting contraceptive use among urban adolescents and youth in Guinea. The objective of this study was to explore the factors that promote the use of contraceptive methods at the personal, interpersonal, community, and health system levels among urban adolescents and youth in Guinea.

**Methods:**

We conducted a qualitative research study including twenty-six individual in-depth interviews among adolescents and youth, and 10 group interviews with an additional eighty individuals, for a total of 106 participants. The socio-ecological model was used to orient both data collection and analysis. Data were collected from June to October 2019. Both individual and group interviews were audio-recorded, and transcribed verbatims afterwards. Data was analyzed thematically, using deductive codes.

**Results:**

The individual factors favoring contraceptive use among adolescents and youth pertained to perceived benefits of the methods (e.g., discretion, absence of side effects, duration of action, ease of use), knowledge of the family planning service channels, and means to afford the cost of the method. The interpersonal factors were spouse/sexual partner approval, and peer suggestions about contraceptive methods. The community factors included socio-cultural beliefs about the methods, and community expectation not to get pregnant before marriage. The health system factors included access to free contraceptive methods, availability of methods, clinical competence and attitude of the health care provider to advise or administer methods, and proximity of family planning services to users’ place of residence.

**Conclusions:**

This qualitative research shows that many adolescents and youth living in Conakry use a variety of contraceptive methods, whether modern, traditional Access to free or affordable methods, discretion of method use, proximity and availability of methods, and suggestions of methods by peers are factors that motivate adolescents and youth to use contraception. In order to optimally facilitate the use of modern contraception among adolescent and young urban Guineans, we recommend that: (1) adolescents and youth have access to public health strategies enabling them to learn about, obtain, and use methods in a way that allows them to remain discreet; (2) the use of modern contraceptive methods be promoted by peers; and (3) health care providers and peers be adequately trained to have accurate and up-to-date knowledge about the different contraceptive methods available, demonstrate clinical skills for teaching and for method placement (if applicable), and show appropriate attitudes toward this population. This knowledge can inform policies and programs to improve the use of effective contraceptive methods by adolescents and youth living in urban Guinea.

## Plain language summary

**Background:**

The use of Family planning (FP) among adolescents and youth is essential to prevent unintended pregnancies. The objective of this study was to understand the personal, interpersonal, community, and health system factors that encourage the use of FP among urban adolescents and youth in Guinea.

**Methods:**

We conducted a qualitative study with adolescents and youth living in Conakry, Guinea. The study took place between June and October 2019. Data were collected through 26 individual in-depth interviews (IDIs), and 10 group interviews (FGDs). Both IDIs and FGDs were recorded and transcribed. Data was analyzed using the “thematic analysis” method.

**Results:**

The personal factors favoring the use of FP among adolescents and youth were related to perceived benefits of FP, knowledge of the FP service availability, means to afford the cost of FP. The interpersonal factors included spouse/sexual partner approval and friends’ suggestions about FP. The community factors included socio-cultural beliefs about the methods, and community expectations not to get pregnant before marriage. The health system factors pertained to access to free FP, availability of methods, clinical competence and attitude of the health care provider, and proximity of FP services to users’ place of residence.

**Conclusions:**

This research shows that many adolescents and youth living in Conakry use a variety of contraceptive methods, whether modern (implant; intrauterine device; injectable; pills; condom; lactational and amenorrhea method), traditional (fixed-day method; abstinence; withdrawal method). In order to optimally facilitate the use of modern contraception among adolescent and young urban Guineans, we recommend that: (1) adolescents and youth have access to public health strategies enabling them to learn about, obtain, and use methods in a way that allows them to remain discreet; (2) the use of modern contraceptive methods be promoted by peers; and (3) health care providers and peers be adequately trained to have accurate and up-to-date knowledge about the different contraceptive methods that exist, demonstrate clinical skills for teaching and for method placement (if applicable), and show appropriate attitudes towards this population. This knowledge can inform FP programs to improve the use of effective contraceptive methods by adolescents and youth in urban Guinea.

## Background

In lower-income countries, approximately 21 million adolescents between 15 and 19 years of age become pregnant each year, and about half of these pregnancies are unintended [1]. In sub-Saharan African countries, the fertility rate among adolescents is the highest globally, averaging 120 per 1000 in 2010 [2]. This high rate is alarming, as the occurrence of pregnancy and childbirth during adolescence carries significant risks of deleterious physical consequences for the mother, such as a higher risk of eclampsia and infections as compared to older women [3, 4]. Moreover, complications related to pregnancy and childbirth are the main cause of death among adolescent females [1]. Unintended adolescent pregnancy has serious socioeconomic consequences, causing many pregnant girls to leave school prematurely, preventing them from reaching a level of education that would allow them to access well-paying employment and have more power over their lives [5]. Furthermore, as sex before marriage is often considered shameful in many sub-Saharan African contexts, unmarried girls who become pregnant may face community stigmatization or family abandonment with the subsequent risks of psychological distress [1, 6, 7] and withdrawal of financial support [8, 9]. Unintended pregnancies negatively affect women of all ages and marital status, as well as their infants, as they are correlated with an increased risk of unsafe abortions, maternal mental health problems (e.g., depression or child-rearing stress), poorer adherence to evidence-based maternal behaviors (e.g., less breastfeeding), and higher infant mortality [10]. Adolescents and young women (aged 15–24 years) living in lower-income countries—including in sub-Saharan Africa—are particularly likely to be affected by unintended pregnancies [11]. Thus, to achieve the sustainable development goal (SDG) of information and education + adolescents/youths by 2030, more work needs to be done to prevent unintended pregnancies among this vulnerable subpopulation [12].

Modern contraception methods are an effective means to prevent pregnancy, but contraceptive use among adolescents and youth remains very low in lower-income regions [13]. Adolescents and youth seeking to use contraceptive methods often face mistrust or rejection by health care providers for their age [14] as well as general difficulties accessing contraceptive services [15]. However, the World Health Organization (WHO), in its medical eligibility criteria for the use of contraceptive methods, explicitly states that: “Age alone is not a medical reason for denying a method to an adolescent” [13].

Qualitative studies on the barriers to meeting the contraceptive needs of adolescents in Nigeria, the Democratic Republic of the Congo and South Africa suggest paying attention to their social, political, cultural and religious environment; their access to health services, the quality of health services; and the cost of contraception [16-18]. The social context for adolescents may include barriers to accessing contraceptive care such as fear or embarrassment of meeting a relative in family planning services and a lack of knowledge of contraceptive methods that limit their use of modern contraceptive methods [5]. Population increases and rapid urbanization in sub-Saharan African cities may further impede health services of urban youth because of the low adaptive capacity of health systems [19].

In Guinea, available modern contraceptive methods include male and female sterilization, injectables, the intrauterine device (IUD), birth control pills, implants, the male and female condom, the Standard Days Method (SDM), and the morning-after pill. Common traditional methods include the lactational amenorrhea (LAM), the periodic abstinence and withdrawal method, and the so-called folk methods, such as herbs, teas and other methods that may also fit into this category [20]. The median age at first sexual intercourse is 16.6 years for women, and 20.5 years for men [20]. Sixty-six percent of females and 23% of males aged 15–19 are sexually active [20]. Moreover, the fertility rate is high among adolescents and young adults, at 146 per 1000 women aged 15–19, and 207 per 1000 women aged 20–24 [21]. The modern contraceptive prevalence remains low and reflects only a slight increase over the past two decades among adolescents and young adults, from 8.4% in 1999 to 12.8% in 2018 [19], demonstrating unmet need and risk of unintended pregnancy [20]. Barriers to contraceptive use identified among adolescents and youth living in Conakry, the capital of Guinea, included the cost of contraceptive methods, fear of side effects, partner’s negative opinion of contraceptive use, religious proscription of contraceptive use, and perceived negative attitudes of health care providers [22].

While knowledge of the factors that impede the use of modern contraception is important, it is also essential to gain knowledge of the factors that can facilitate and promote their use to better inform public health strategies. However, to our knowledge, no study has explored and documented the factors facilitating the use of contraception among urban adolescents and youth in Guinea. The objective of this study was therefore to explore the personal, interpersonal, community, and health system factors that promote the use of contraceptive methods among urban adolescents and youth (female and male) living in Conakry, Guinea. As a side note, we specifically focused on facilitators in this paper, as we have already reported on barriers in another paper published elsewhere [22].

## Methods

### Study design

This qualitative study used an exploratory design [23]. Data were collected from June to December 2019 among adolescents (15–19 years old) and youth (20–24 years old) living in the urban setting of Conakry.

### Study framework

The socio-ecological model [23] was used to structure the categories of concepts that were explored and framed the analysis of the data. This conceptual framework proposes several levels of factors that influence health behaviors. Thus, we considered the following factors across the multiple levels to explore the facilitators promoting the use of contraceptive methods among urban adolescents and youth: (1) individual factors, such as participants’ perceived benefits of a contraceptive method, their knowledge of contraceptive service channels, and their ability to afford the cost of the contraceptive method; (2) interpersonal factors, such as the influence of boyfriend/spouse, peers or parents; (3) community factors, such as socio-cultural beliefs; and (4) health systems factors, such as quality, availability, accessibility, and proximity of services for contraceptive methods.

### Study setting

**Fig. 1:**
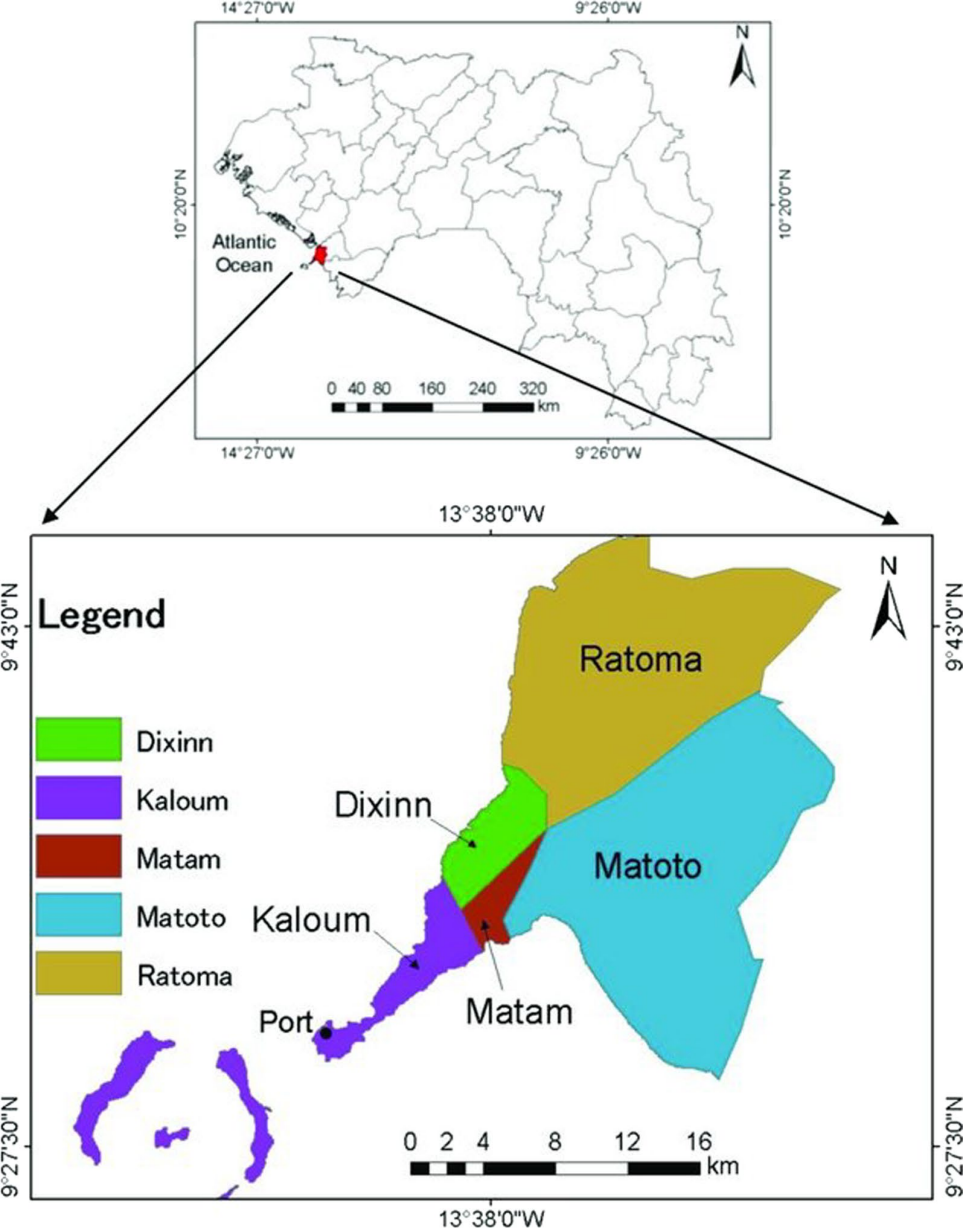
Map of the region of Conakry and its five communes in Guinea [25]. Source Land-cover change analysis and simulation in Conakry (Guinea), using hybrid cellular-automata and Markov model (2018)

The study took place in Conakry, the capital city of Guinea. Guinea is a country located in western Africa on the Atlantic Ocean, and has a population of 12,907,395 inhabitants [24]. One-fifth of the population is adolescent (15–19 years old, 10.6%) or young adult (20–24 years old, 8.8%) [24]**.** The country is composed of eight administrative regions, including Conakry, the capital city. The population of Conakry is estimated at 1,984,767 inhabitants, representing 16% of the national population [24]. Adolescents and youth represent 55.5% of Conakry’s population [24], making them an important subpopulation for which special attention should be given. The Conakry region is an urban area comprising five communes—Kaloum, Matam, Dixinn, Matoto, Ratoma—(Fig. 1), further divided into 130 neighborhoods.

### Study participants and recruitment

Data were collected from 26 adolescents and youth aged 15–24 living in the urban setting of Conakry between June and October 2019. Both genders were included as women are the primary users of contraceptive methods and bear the burden of pregnancy and pregnancy-related health problems, yet men have an influence on women's contraceptive decision-making and are key decision-makers for the use of male condoms. Moreover, involvement of both members of a couple in sexual and reproductive health programming can promote the adoption and use of contraceptive methods in patriarchal societies [26]. Inclusion criteria included aged 15–24 years old and live in one of the five communes of Conakry; no other exclusion criteria were applied. Adolescents and youth could be users or non-users of contraception methods; single, in a relationship, or married; and in or out of school. Participants were purposively selected for diversity in socio-demographic characteristics (i.e., gender, level of education, and marital status) in order to capture a variety of experiences and perspectives. They were recruited through the administrative hierarchy of each commune, i.e., the mayor, the district and sector chiefs. In each commune, three neighborhoods were selected based on the availability of the neighborhood heads and the perceived ease of access to adolescents and youth. Within each neighborhood, three sectors were randomly selected by the data collection agents. Adolescents and youth in each sector were put in contact with the interviewers by the sector heads. Those who consented or assented to participate in the study (see “Ethical considerations” subsection) were invited to schedule an interview at a time convenient to them. Interviews were conducted in private settings to ensure confidentiality.

### Data collection

Data were collected through two modes: individual in-depth interviews (IDIs) and focus group discussions (FGDs). Individual interviews allowed for thorough exploration of participant's personal stories. This mode is also more appropriate for discussing personal or taboo topics such as sexuality. Focus group discussions allowed data collectors to stimulate discussion among participants as well as to enrich the findings from individually-collected data. The individual and group interview guides incorporated the following domains: previous and current contraceptive use and experience; general knowledge about the contraceptive method participants use (e.g., advantages and disadvantages); attitudes of relatives about contraception methods; experience with contraceptive services (including with contraceptive service providers); and participants’ demographic and socioeconomic characteristics (age, marital status, education, occupation and commune of residence).

All interviews were conducted by a team of experienced and trained qualitative interviewers (young public health physicians and sociologists). Interviews took place in the locations chosen by the participants and were conducted in French or a local language of the participants’ choice (Soussou, Malinké or Peulh). The interviews were audio-recorded with participants’ permission. Notes were duly taken for participants who did not consent to an audio recording of their interview. The total sample size was 106 participants. Twenty-six [26] IDIs were conducted. Two FGDs were held per commune, separately gathering eight adolescent and young women and eight adolescent and young men, for a total of 80 participants across 10 FGDs. Average length of the IDIs was 45 min while FGDs lasted 1h30min in average. All data collected were managed at the National Rural Health Training and Research Center (NRHTC) of Maferinyah (Guinea), where survey forms, recordings, and interview transcripts were kept in a locked cabinet.

### Data analysis

IDI and FGDs were transcribed and translated into French as needed. The data analysis was conducted by the research team using transcripts and IDI/FDG notes. Coding was done in excel using the “thematic content analysis” method [27]: deductive coding was performed using themes found in the literature review and building on the IDI and FGD guides [28].

The data were coded by trained researchers (HM, ND, and CB), Each of whom analyzed the transcription. After familiarization with the content of each transcript, each researcher developed codebooks on Excel sheets. These codebooks were then compared for similarities, discrepancies, and complementarity at two discussion meetings including the other co-authors. A consensus codebook was then adopted and guided the parallel coding of the data. Information items relevant to the research questions were highlighted in the Word document and coded using the “new comment” option. The highlighted information items were then copied and pasted into an Excel sheet with the name and short definition of the respective codes, as well as the ID number and characteristics of the respondents. Next, the codes were organized into themes and subthemes, taking into account similarities and differences, using the Excel sheets. Each coder interpreted the themes/subthemes and identified illustrative quotes. The interpretation of the themes/subthemes and the corresponding illustrative quotes were discussed in a third meeting with all co-authors.

### Trustworthiness

We used various techniques to ensure the rigor of analyses and the credibility of results. We coded the data in a systematic and consistent manner and organized data summaries in data reduction tables housed in a Microsoft Excel spreadsheet [29]. Excerpts from the participants' interviews (quotes) were included in the Results section of the document, so that readers could have confidence in the interpretations. Finally, the interpretations were validated by the research team [30]. Prior literature from other research in sub-Saharan Africa was used to validate the results [31]. We incorporated participant narratives in the current scientific paper to support our interpretations [31].

### Reporting

We used the SRQR reporting guidelines [32].

### Ethical considerations

The research protocol was approved by the National Committee of Ethics for Health Research of Guinea (CNERS). The interviewers led potential adult participants (18–25 years of age) through an informed consent process, including an introduction to the study and a discussion of the risks and benefits of study participation. Participants that were minors (aged 15–17 years) were asked for their assent to participate, and their parents/guardians were asked for their consent for the participation of their child/dependent in the study. All participants provided written confirmation of informed consent/assent before participating in the study. Anonymity of participants was ensured in the presentation of research findings.

## Results

### Sociodemographic characteristics of participants

The characteristics of the 26 adolescents and youth that were individually interviewed, as well their communes of residence, are shown in Table 1. The majority of them were female (65.4%) and had attended formal school (61.5%). More than half were married (53.8%). The characteristics of the 80 focus group participants are described in Table 2.

**Table 1:** Characteristics of participants in individual in-depth interviews

Socio-demographic characteristics	N (n = 26)	Percentage (%)
Types of participants		
Adolescents (15–19)	9	34.6
Youth (20–24)	17	65.4
Sex		
Men	8	30.8
Women	18	69.2
Commune of residence		
Kaloum	6	23.1
Dixinn	6	23.1
Matam	4	15.4
Ratoma	5	19.2
Matoto	5	19.2
Schooling		
Attended formal school	16	61.5
Did not attend formal school	10	38.5
Marital status		
Married	14	53.8
Single	12	46.2

**Table 2:** Characteristics of participants in focus group discussions

Socio-demographic characteristics	Number of FGDs (total: 10)	Number of participants (n = 80)
Types of participants		
Adolescents (15–19)	5	40
Youth (20–24)	5	40
Sex		
Men	5	40
Women	5	40
Commune of residence		
Kaloum	2	16
Dixinn	2	16
Matam	2	16
Ratoma	2	16
Matoto	2	16
Schooling		
Attended formal school	7	56
Did not attend formal school	3	24
Marital status		
Married	6	48
Single	4	32

### Contraceptive methods use among urban Guinean adolescents and youth

According to participant narratives, different modern and traditional contraceptive methods are used by urban Guinean adolescents and youth. The modern methods include the implant, the intra uterine device (IUD), the injectable method, the oral contraceptive (the “pill”), the emergency contraception “morning-after pill”, female and male condoms, the lactational amenorrhea method (LAM), and the fixed-day method (FDM)—also known as the “cycle necklace”. The traditional methods used by some of the participants include abstinence and the withdrawal method (coitus interruptus).

### Factors promoting the use of contraceptive methods among urban Guinean adolescents and youth

Participants’ accounts about the factors that encourage them to use contraceptive methods were categorized into four main themes: personal, interpersonal, community, and health system factors, which are presented below.

#### Personal factors promoting contraceptive use

Three factors related to the individuals themselves were identified in the data as promoting contraceptive use, regardless of age group: participants’ perceived benefits of a contraceptive method, their knowledge of contraceptive service channels, and their ability to afford the cost of the contraceptive method.

#### Perceived benefits of contraceptive methods

Most unmarried participants reported using contraceptive methods to avoid pregnancies outside of marriage, which are considered a source of dishonor for Guinean families.

*“For my part, I use the implant to avoid bringing shame to my family because in our country, if you are not married and you get pregnant, it is a shame for your family. It will be very badly judged by the community”* (Young unmarried woman commune of Dixinn).

Participants who already have children explained that they use contraception to space births.

Contraceptive method benefits perceived by adolescents and youth varied from one method to another. These benefits included discretion, absence of side effects, duration of action, ease of use, dual protection against unintended pregnancies and sexually transmitted infections (including human immunodeficiency virus), and body appearance improvement based on the perception that a method can increase user’s weight in case she feels too thin. These are described in the following section.

Discretion was an important benefit reported by adolescents and young people for choosing a contraceptive method, as it allows for use without their parents being aware of it, thus limiting the need to disclose they have started to have sexual intercourse. This was the case for the condom, the injectable method, as well as for the implant.

“*My parents are very strict, that’s why I prefer the implant and I use it because when you place the implant your parents will never know it. Also, at home I wear long sleeves so that the scar does not show.”* (IDI, young woman, single, commune of Ratoma).

Use of the morning-after pill and the condom was largely motivated by the lack of side effects, such as menstrual disorders and fear of infertility attributed to other methods.

“*... with this method [morning-after pill], menstruation is not blocked”* (FGD young woman, single, commune of Matam).

*“Because of the fact that the injectable method and also the implant block menstruation, I am afraid to stay without getting pregnant when I am married”* (IDI, unmarried female adolescent, commune of Ratoma).

Some users reported preferring methods with long duration of action, including both modern and traditional methods. This was the case with the injectable method, implants whose duration of action ranges from 3 months for the injectable method. According to users, their preference for a long-acting contraceptive method is motivated by not having to visit a health care provider as often as with other contraceptive methods. In their opinion, frequent visits for contraceptive services consume time and money for transportation, and expose them to social stigma as the use of (modern) contraceptive methods is “forbidden by religion” or by their parents.

“*I often use the implant, which is valid for 5 years, so I don’t have to go to the hospital every time to ask for contraceptives”* (IDI IDI, Young married woman, commune of Matoto).

In addition, participants reported that condom use is motivated by two main reasons: its ease of use that does not require the intervention of a health care provider, and its effectiveness in also preventing STIs/HIV.

*“Because the [condom] can prevent not only unintended pregnancies, but also dangerous diseases such as AIDS”* (IDI, unmarried male adolescent, commune of Matoto).

Finally, the benefit of improved body appearance was especially mentioned by young, frail or overly thin women who wish to gain weight using the depo-provera injection.

*“There are young girls who say, I’m so thin, I’m [advised] [to use the depo-provera injection] ... I would like to put on some weight”* (IDI, unmarried female adolescent, commune of Matoto).

#### Knowledge of the FP service channels

Participants stated that their familiarity with the health facility allows them to use FP services discreetly without being seen by people they know or having to ask strangers for information about the service requested.

*“At the CMC [medico-surgical center] Bernard Kouchner, it’s easy for me to get [the contraceptives], I know the service where the doctor who does [the injection] is. When I come, I go directly there, no one sees me and because of that, I use [contraception] regularly”* (IDI, Young married woman, commune of Kaloum).

#### Means to afford the cost of the contraceptive method

The use of the contraceptive method is also promoted by user’s capacity to withstand the cost. For example, despite their preference for implants, which is a discreet and long-acting method, some adolescents and youth said they use pills because they find them more affordable (5000 to 10,000 GNF—50 cents to 1 euro—for a duration of 6 months). However, other adolescents and youth used the implant stating they considered it as affordable (100,000 to 150,000 GNF—10 to 15 euros—for a duration of 2 to 5 years).

*“... earlier [I had used the implant], but [now], as I couldn’t afford it, I use pills”* (IDI, young woman, single, commune of Kaloum).

*“I prefer [the implant], it is not expensive. That’s what’s good for me. I manage to pay without any problem”* (IDI, young woman, single, commune of Dixinn).

### Interpersonal factors promoting contraceptive use

In this study, two categories of interpersonal factors favoring contraceptive use were cited by urban adolescents and youth in Conakry: spouse/sexual partner approval, and peer opinions (friends, classmates, co-workers).

#### Spouse/sexual partner approval

The favorable opinion of the spouse or sexual partner also appeared to be a factor facilitating the use of a family planning method, especially since culturally, it is generally the men who have more power within Guinean couples. Indeed, according to participants, some spouses agree that their wives use a contraceptive method to space births, and others may even require it. Some also stated contraception is good for the woman’s health.

*“My husband sent me to the hospital to have the implant placed in my arm because I had complications with the birth of my last son and he thought that the implant was better [to avoid getting pregnant again quickly]. According to him, when we are ready, we can remove it”* (IDI, young married woman, commune of Matoto).

#### Peer suggestions for contraceptive methods

Adolescents and youth, regardless of gender, emphasized that peer suggestions about a contraceptive method motivate their use of that method. These suggestions are usually made as advice by peers who have used the method before and have had a positive experience with it.

*“I have a friend who told me that the implant is a good method, which protects against pregnancy. Since then, I have been using the implant; otherwise, I didn’t know anything about contraceptives”* (IDI, unmarried adolescent, commune of Matoto).

### Community factors promoting contraceptive use

#### Socio-cultural beliefs about the methods

Some participants referred to certain socio-cultural beliefs about particular contraceptive methods, *Community expectation not to get pregnant before marriage.*

In addition, not getting pregnant outside of marriage is an expectation in communities, as it is a source of dishonor for girls and their families, and may even prevent a girl from finding a husband.

*“In our neighborhood here, our mothers force us to use the abstinence method to prevent us from getting pregnant, because [getting pregnant while unmarried] is strictly forbidden in our community. If you get pregnant, men refuse to marry you”* (FGD, unmarried adolescent, commune of Kaloum).

### Health system factors promoting contraceptive use

Based on what we found to be most salient in participants’ narratives, the health system factors that most supported contraceptive use were: access to free methods, availability of the method, clinical competence and attitude of the contraceptive service provider, and proximity of contraceptive services to users’ homes.

#### Access to free contraceptive methods

According to adolescents and youth who use contraceptive methods, some health facilities organize awareness campaigns with free distribution of contraceptive methods. They stated that the effectiveness of the messages conveyed during these campaigns and above all the fact that the methods are free favor the use of these methods promoted by health care providers.

*“There is an awareness campaign on family planning at the health center here [Centre médico-chirurgical Bernard Kouchner], we are happy because we are given the methods we want for free”* (IDI, unmarried adolescent, commune of Kaloum).

#### Availability of contraceptive methods

The availability of contraceptive methods (e.g., in health facilities or drug stores) favors their use by adolescents and youth. Indeed, this motivates adolescents and youth to use those methods since they can easily obtain them when needed. This is the case for condoms that are sold in pharmacies and neighborhood stores.

*“... condoms can be bought everywhere, you can even find them in stores”* (FGD adolescent, single, commune of Dixinn).

#### Clinical competence and attitude of the FP service provider

Implant users emphasized the importance of the provider's clinical competence and attitude, which includes discipline, discretion, understanding and motivation providers in encouraging them to use this method. They felt that pre-placement counseling and painless incision for placement of the method was a quality service that promoted contraceptive use.

*“The doctors at this center do their job very well. First of all, before moving on to anything else, they sensitize the client, making her understand whether what they are doing is good or not, they put it in well and it doesn’t hurt at all”* (IDI, young woman, commune of Matoto).

In addition, the majority of modern contraceptive users find that the contraceptive service provider’s attitude toward the client is a promoting factor for method use. In their view, it is important that there be a good relationship between contraceptive providers and clients. Moreover, they value that providers respect confidentiality and make no distinction based on the marital status of clients, i.e., that they do not discriminate against unmarried adolescents and youth that are sexually active and seeking contraceptive methods.

#### Proximity of contraceptive services to users’ place of residence

Proximity to health facilities offering contraceptive services was reported by adolescents and youth as a promoting factor for contraceptive use.

*“The health center is close to my home, so it’s easy for me to get injected with the depo-provera, I can go at any time”* (IDI, young unmarried woman, commune of Matoto).

## Discussion

Our study reveals that urban Guinean adolescents and youth use diverse contraceptive methods. These methods include modern short and long-acting methods which generally have highly effectiveness if correctly used, traditional methods including fixed-day method, abstinence, and withdrawal method, whose effectiveness is lower and conditional to correct usage [33],

This research describes factors that promote contraceptive use among urban adolescents and youth (15–24 years old) living in urban Guinea. Individual-level factors enabling the use of these methods include perceived benefits of the methods (i.e., discretion, absence of side effects, duration of action, ease of use, dual protection against unintended pregnancies and STIs/HIV, and body appearance improvement), knowledge of family planning service channels, and ability to pay for the method. Interpersonal factors favoring contraceptive use are spouse or sexual partner approval, and peer opinions/suggestions. Community factors relate to some socio-cultural beliefs about the methods and community expectation not to get pregnant before marriage. Health system factors include access to free methods, availability of the method, contraceptive service provider’s competence and attitude, and proximity of contraceptive services. Put differently, our data show that urban adolescents and youth living in Conakry wanting to use contraception mainly base their decision to use a specific method according to the following domains: (1) perceived benefits of the method (2) easy procurement, and (3) influence of key actors of significance to them. These findings are discussed below with the aim of making recommendations to improve the promotion of effective contraception use among adolescents and youth in Conakry.

### Facilitating the use of modern contraception methods by focusing on the preferences of adolescents and young people

Our findings support that a user’s perceived benefits of a contraceptive method promote its use. Strategies aimed at promoting the use of modern contraceptive methods should therefore take into account the preferences of adolescents and youth in order to increase the likelihood that they can effectively achieve their reproductive goals.

#### Access to free or affordable methods

To maximize contraceptive use among adolescents and youth in the urban Guinean context, it is important to consider the affordability of the recommended contraceptive methods. Our study revealed that affordability of contraceptive methods represents a key transversal individual and health system factor influencing contraceptive use among adolescents and young people in Conakry, as many respondents mentioned that they are keen to use methods when they are free of charge or at a low cost. This can be explained by the social configuration in Guinea, where adolescents and youth are generally economically dependent on their parents, but most of them refrain from seeking money from their parents to pay for contraceptive services [22, 34]. Indeed, the use of contraceptive methods is perceived by some parents as socially deviant, as it implies sexual intercourse among unmarried people [34]. Therefore, the cultural norm of abstinence from premarital sex which is related to the preservation of family honor makes many unmarried adolescents and youth seek to conceal from their parents the fact that they are sexually active [22, 35, 36]. Contraceptive use is also believed to encourage infidelity for married people, which is not recommended by religion [34]. In addition, sexuality and contraception are strong taboo topics in the Guinean sociocultural context, particularly between parents and children but also often within couples [22]; talks on these issues are therefore not encouraged within families.

Recognizing these socio-cultural sensitivities, the health system should find ways to provide contraceptive methods free or at low-cost, for example by subsidizing contraceptive services, to support adolescent and youth access regardless of their family's socioeconomic level. However, the operationalization of such policies is very challenging in resource-constrained settings such as Guinea [37]. Nevertheless, alleviating financial barriers to improve adolescents and youth access to contraceptive methods should be an integral part of strategies to reduce unmet need these age groups.

Moreover, long-acting methods (e.g., implant) are also more expensive at the outset, but their renewal is spaced out, as opposed to methods that need to be renewed more often (e.g., the pill). It may therefore be appropriate for contraceptive counselors to reorient their presentation of method costs to help adolescents and youth see the overall cost of each method (e.g., per year) so they can make an informed decision about the fees, as well as to offer the possibility of payment in installments, if possible.

#### Discretion of method use

Discretion appeared to be a very important characteristic for use of specific contraceptive methods among the population of adolescents and youth living in the socio-cultural context of Conakry, especially among young women. This was indeed cited explicitly by participants, as well as implicitly through their desire for free supplies (as noted above), their preference for long-acting contraceptive methods which allow them to avoid being seen too often at the contraceptive clinic, and their preference for familiar health facilities which allows them to use contraceptive services discreetly. Again, through their search for discretion, adolescents and young people show that they want to conceal their use of contraceptive methods to their parents and from other people around them, as sexual activity is forbidden outside of marriage, or is a taboo subject. Additionally, because of the power differential between men and women in Guinea where men often have the ultimate decision-making power [38, 39], there may be a desire on the part of women to be discreet about their contraceptive use in case of non-approval/non-support from their spouse or sexual partner. This phenomenon was also observed in a study carried out in three sub-Saharan African countries (Ethiopia, Nigeria, Uganda), where it was found that women hide from their spouses to use contraceptive methods to space births, which the spouses dislike because they want more children [40].

Some participants pointed out their preference for using condoms, that they found easier to use than other methods as it does not require the intervention of a health care provider. This can be interpreted as discretion, as youth want to avoid disclosing that they are having sexual intercourse, even to health care providers. In addition, women hide their use of contraceptive methods for fear of affecting their relationship with their partner if he is against contraception.

In sum, these factors mean that adolescents and youth who want to be sexually active while preventing unintended pregnancies should have access to public health strategies enabling them to learn about, obtain, and use methods in a way that allows them to remain discreet. Those promoting and supporting the use of contraceptive methods (e.g., Ministry of Health, health facilities, health professionals, NGOs) should therefore take the need of discretion into account when developing/revising their public health strategy.

#### Prevention of HIV/AIDS

A number of participants, particularly men, expressed the view that the use of condom is not only a protection against unintended pregnancies, but also against HIV/AIDS. In Guinea, 83% of adolescents and 85% of young people know about HIV/AIDS, and that using condoms during sexual intercourse can protect them from this serious infection [20]. Nevertheless, ensuring awareness among adolescents and youth about preventive measures for sexually transmitted diseases, including HIV/AIDS, could contribute to their well-being.

#### Other perceived benefits

Also, some women stated they find that weight gain following contraceptive use is a factor that motivates its use. This suggests that some adolescents and youth do not use modern contraceptive methods only for pregnancy prevention, but also for their perceived effect on body appearance. A study conducted in Ghana [41] revealed that the adoption and use of certain contraceptive methods lead to weight loss and was therefore a demotivating factor in the adoption of contraception. Weight gain or loss is seen as one of the unwanted side effects of contraception, particularly pills, and therefore a barrier to its use***.*** Training health personnel involved in contraceptive service delivery on sex education and sensitizing adolescents and youth during medical appointments could help to eliminate misconceptions and ultimately help them to identify the most appropriate methods with fewer negative effects.

### Facilitating the use of modern contraceptive methods by improving adolescents’ and youth’s access to supplies

#### Proximity of FP services and availability of methods

Adolescents and youth expressed the need to have an easy access to contraceptive methods, in terms of proximity to supply points and of product availability. Participants prefer this proximity mainly because of the difficulty of moving around Conakry efficiently. Indeed, the public transport network is almost non-existent, and traffic jams are recurrent; they do not want to spend too much time traveling so they can do the activities they like. The ease of access was indeed cited by some participants for choosing specific products. Understanding youth preferences for readily available methods can inform counseling interventions to guide the adoption of modern methods that are easily available, in addition of being scientifically proven to be effective and efficient.

### Facilitating the use of modern contraceptive methods among adolescents and youth by supporting the involvement of appropriately trained key actors

According to our participants’ narratives, information on contraceptives could be delivered by health care providers, but also by peers.

#### Promotion by health care providers

Health care providers are central actors in contraceptive counseling and support. According to our participants, providers must be competent in counseling skills as well as clinically, such as for the placement of the contraceptive method (when it applies). They also emphasized the importance of providers having a non-judgmental attitude towards them and respecting confidentiality, which is consistent with recommendations of the Lancet commission on adolescent health and wellbeing [41]. In order to be able to do so, health care providers should be adequately trained to (1) have accurate and up-to-date knowledge about the different contraceptive methods that exist, their advantages, disadvantages or undesirable side effects, and on their use; (2) demonstrate clinical skills for teaching and for method placement (if applicable); and (3) show appropriate attitudes with this population [41].

#### Promotion by peers

Our findings have illustrated that peer suggestions for contraceptive use have led to some adolescents and youth utilizing a particular contraceptive method. We thus argue that a peer education model involving adolescent and youth contraceptive users in contraceptive counseling may be a potentially effective way of reducing unmet contraceptive needs among this social stratum, provided that they are properly trained and supported.

Adolescence is indeed a time when youth expand their circle of influence beyond the family, and place great importance on the opinions of their peers [41]. Adolescents and youth can have a significant influence on the decisions of their peers, including on behaviors that affect health and well-being such as sexual and reproductive health, influence that can either be positive or negative [41]. In their review of the literature, Patton et al. [41] found that peer education, defined as education provided by adolescents and youth to others in their age group, can be an effective strategy in low-income countries for promoting safe sexual behaviors, including contraception use. We recommend that such a peer education strategy for contraception be developed and tested for its effectiveness in facilitating access to modern contraception methods among Guinean adolescents and youth.

### Strengths and limitations of the study

To our knowledge, this is the first study to explore the factors that facilitate contraceptive use by adolescents and youth living in the city of Conakry in Guinea. The strengths of this study lie in the fact that we captured a wide range of respondents: adolescents and youth, literate and illiterate, married and single, young men and women. This allowed for a wide variety of personal experiences and/or perspectives that contributed to provide a nuanced understanding of the topic under study. On the other hand, our study sample was limited to only urban adolescents and youth living in Conakry: we therefore cannot conclude that these findings apply to adolescents and youth who live in other cities and rural or forest areas of the country.

## Conclusion

This qualitative research shows that many adolescents and youth living in Conakry use a variety of contraceptive methods, whether modern, traditional or based on socio-cultural beliefs. In order to optimally facilitate the use of modern contraception among adolescent and young urban Guineans, we recommend that: (1) adolescents and youth have access to public health strategies enabling them to learn about, obtain, and use methods in a way that allows them to remain discreet; (2) the use of modern contraceptive methods be promoted by peers; and (3) health care providers and peers be adequately trained to have accurate and up-to-date knowledge about the different contraceptive methods available, demonstrate clinical skills for teaching and for method placement (if applicable), and show appropriate attitudes toward this population.

This knowledge can inform policies and programs to improve the use of effective contraceptive methods by adolescents and youth in Guinea, and ultimately to contribute to optimal sexual and reproductive health of this population.

## Data Availability

Access to the data used in this analysis may be obtained upon reasonable request from the authors.

## Abbreviations

FP: Family planning
FGDs: Focus group discussions
IDIs: In-depth interviews
HIV: Human immunodeficiency virus
AIDS: Acquired immune deficiency syndrome
STI: Sexually transmitted infection
